# Guideline on the use of onabotulinumtoxinA in chronic migraine: a consensus statement from the European Headache Federation

**DOI:** 10.1186/s10194-018-0921-8

**Published:** 2018-09-26

**Authors:** Lars Bendtsen, Simona Sacco, Messoud Ashina, Dimos Mitsikostas, Fayyaz Ahmed, Patricia Pozo-Rosich, Paolo Martelletti

**Affiliations:** 1 Danish Headache Center, Department of Neurology, Rigshospitalet-Glostrup, Faculty of Health and Medical Sciences, University of Copenhagen, 2600 Glostrup, Denmark; 2 Neuroscience Section, Department of Applied Clinical Sciences and Biotechnology, University of L’Aquila, 67100 L’Aquila, Italy; 3 1st Department of Neurology, Aeginition Hospital, National and Kapodistrian University of Athens, Athens, Greece; 4 Department of Neurosciences, Hull York Medical School, Hull, UK; 5 Headache & Craniofacial Pain Unit, Neurology Department, Hospital Universitari Vall d’Hebron, Barcelona, Spain; 6 Headache Research Group, Vall d’Hebron Research Institute (VHIR), Universitat Autònoma de Barcelona, Barcelona, Spain; 7 Department of Clinical and Molecular Medicine, Sapienza University, Rome, Italy

**Keywords:** Chronic migraine, OnabotulinumtoxinA, Management, Guideline

## Abstract

OnabotulinumtoxinA is being increasingly used in the management of chronic migraine (CM). Treatment with onabotulinumtoxinA poses challenges compared with traditional therapy with orally administered preventatives. The European Headache Federation identified an expert group that was asked to develop the present guideline to provide recommendations for the use of onabotulinumtoxinA in CM. The expert group recommend onabotulinumtoxinA as an effective and well-tolerated treatment of CM. Patients should preferably have tried two to three other migraine prophylactics before start of onabotulinumtoxinA. Patients with medication overuse should be withdrawn from the overused medication before initiation of onabotulinumtoxinA if feasible, if not onabotulinumtoxinA can be initiated from the start or before withdrawal. OnabotulinumtoxinA should be administered according to the PREEMPT injection protocol, i.e. injecting 155 U–195 U to 31–39 sites every 12-weeks. We recommend that patients are defined as non-responders, if they have less than 30% reduction in headache days per month during treatment with onabotulinumtoxinA. However other factors such as headache intensity, disability and patient preferences should also be considered when evaluating response. Treatment should be stopped, if the patient does not respond to the first two to three treatment cycles. Response to continued treatment with onabotulinumtoxinA should be evaluated by comparing the 4 weeks before with the 4 weeks after each treatment cycle. It is recommended that treatment is stopped in patients with a reduction to less than 10 headache days per month for 3 months and that patients are re-evaluated 4–5 months after stopping onabotulinumtoxinA to make sure that the patient has not returned to CM. Questions regarding efficacy and tolerability of onabotulinumtoxinA could be answered on the basis of scientific evidence. The other recommendations were mainly based on expert opinion. Future research on the treatment of CM with onabotulinumtoxinA may further improve the management of this highly disabling disorder.

## Introduction

Chronic migraine (CM) is a debilitating disorder affecting approximately 2% of the general population [1] and is very common in specialised headache centres. CM is defined as headache occurring on ≥15 days per month for > 3 months of which ≥8 days has the features of migraine headache [2]. Only two treatments have demonstrated efficacy in CM: onabotulinumtoxinA and topiramate [3].

Possible efficacy of onabotulinumtoxinA in migraine was incidentally noted in patients treated cosmetically for wrinkles. In 2010, onabotulinumtoxinA was reported effective for the treatment of CM in the Phase 3 Research Evaluating Migraine Prophylaxis Therapy (PREEMPT) trials [4, 5] and was approved both by the European Medicines Agency and by the US Food and Drug Administration for the prophylaxis of CM. Its use was endorsed by the National Institute for Health and Care Excellence (NICE) in 2012 [6]. OnabotulinumtoxinA has not been found effective in episodic migraine or in tension-type headache [7]. The mode of action of onabotulinumtoxinA in CM may include modulation of neurotransmitter release, changes in surface expression of receptors and cytokines as well as enhancement of opioidergic transmission [8]. It is likely that onabotulinumtoxinA reduces both peripheral and central sensitization in CM through such mechanisms [7, 9].

Since its approval, the use of onabotulinumtoxinA has increased considerably. Treatment with onabotulinumtoxinA poses challenges compared with traditional therapy with orally administered preventatives for headache specialists not used to injecting toxins. Due to the absence of European Guidelines for the use of onabotulinumtoxinA, the European Headache Federation has considered the need to write a Guideline for the use of onabotulinumtoxinA in CM.

The aim of this guideline is to provide recommendations for the use of onabotulinumtoxinA in CM.

## Methods

The EHF identified an expert panel consisting of seven members. We have developed recommendations for a series of questions that are essential for daily clinical use of onabotulinumtoxinA in CM, which are based on the available evidence and our clinical experience.

We have answered the following questions:

1. Is onabotulinumtoxinA effective and well-tolerated for the treatment of CM?
2. When should onabotulinumtoxinA be offered?
3. Should withdrawal be performed before treatment with onabotulinumtoxinA in patients with CM and medication-overuse?
4. How should the treatment be administered?
5. When can a de novo patient be considered non-responder to onabotulinumtoxinA?
6. How should responders to onabotulinumtoxinA be managed over time?

In the discussion section we have summarized our findings and added some general considerations.

The Grading of Recommendations, Assessment, Development and Evaluation (GRADE) system has been endorsed by the European Academy of Neurology [10] as the method of choice to establish recommendations and was used here if possible as was the Patients; Intervention; Comparison and Outcome (PICO) [10] method. Final quality of evidence was rated as high, moderate, low or very low based on study design, study limitations, inconsistency, indirectness, imprecision, publication bias, effect size, dose response and confounding. Strength (strong or weak) and direction (for or against) of recommendation were determined on basis of balance between desirable and undesirable effects, quality of evidence, values and preferences and costs [10]. With our present scientific evidence this could only be used for the first question. If GRADE was not applicable, an ungraded good practice statement was given, according to the available level of evidence. The Delphi method was used to reach consensus.

## Search strategy and results

Papers published in peer-reviewed journals were identified using PubMed/Medline, EMBASE and Cochrane Library. Search was done with unrestricted date of start and until April 2018 and restricted to English language. We searched for prospective studies investigating efficacy, safety or tolerability of onabotulinumtoxinA in CM compared with placebo or other prophylactic treatments and for prospective observational studies published in peer-reviewed journals. We excluded retrospective studies, studies not performed according to PREEMPT, studies of poor quality, e.g. studies with insufficient reporting of diagnostic criteria or outcome and reviews.

**Fig. 1:**
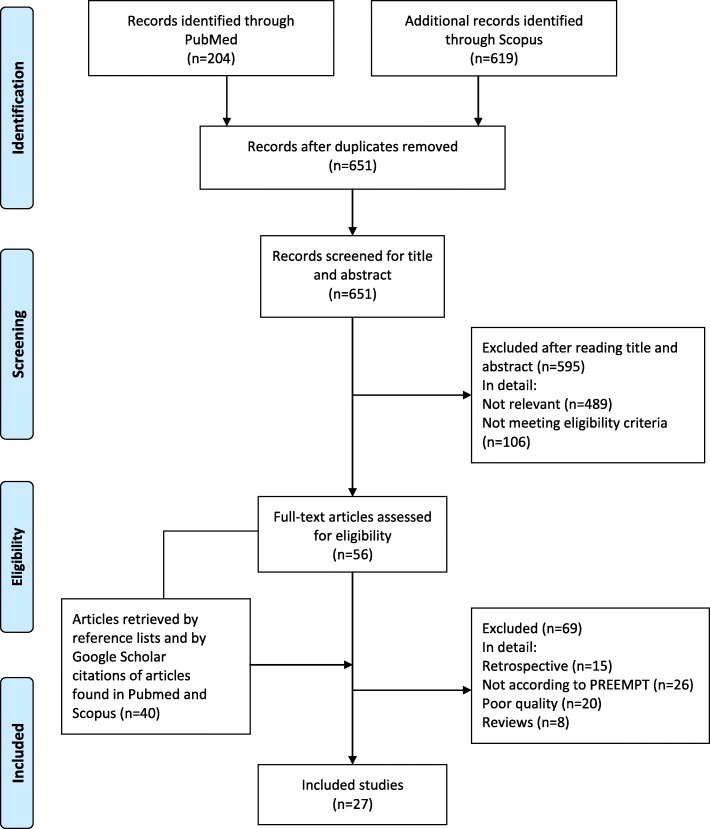
Process of identifying eligible studies for the guideline

We initially identified 823 studies which were finally reduced to 27 studies. Please see Fig. 1. The retrieved studies were all considered for question 1. For the following questions search was performed among the retrieved studies.

## Recommendations

### Question 1: For patients with CM is treatment with onabotulinumtoxinA effective and well-tolerated?

PICO:

- *Population*: patients with CM with or without medication overuse
- *Intervention*: onabotulinumtoxinA 155 U to 195 U
- *Comparison*: placebo or other prophylactic treatments
- *Outcome*: meaningful reduction of headache days (> 30% from baseline) with acceptable side-effects

#### Search strategy and results

We identified two randomized placebo-controlled multicentre trials, the PREEMPT 1 [4] and PREEMPT 2 [5] (Phase 3 Research Evaluating Migraine Prophylaxis Therapy) clinical trials. Both trials consisted of a 24-week placebo-controlled phase followed by a 32-week open-label extension phase. OnabotulinumtoxinA was administered at 12-week intervals. OnabotulinumtoxinA was injected in 31 sites (5 U per injection) with possibility for additional 8 injections according to a “follow-the pain” strategy. The total dose of onabotulinumtoxinA was 155 U to 195 U injected in 31 to 39 sites. This is hereafter referred to as the PREEMPT injection protocol. A total of 1384 patients received onabotulinumtoxinA or placebo. Approximately two-thirds of CM patients were overusing headache medications at baseline. There was no significant effect on the primary endpoint, headache episodes, in the PREEMPT 1 trial, while there was a significant effect on headache and migraine days. The PREEMPT 2 trial met its primary endpoint headache days. The results of the PREEMPT 1 and 2 trials have been evaluated in a pooled analysis [11]. The pooled analysis reported a large decrease from baseline in headache days, but due to a large placebo-effect, the efficacy of onabotulinumtoxinA over placebo was modest (− 8.4 vs. -6.6 days per 4 weeks, *P* < 0.01) [11]. Responder rate, defined as percentage of patients with a decrease in frequency of headache days from baseline of at least 50%, was 47.1% vs. 35.1% (*P* < 0.001). Post-hoc analysis demonstrated that 71.4, 9.4 and 5.4% of patients responded to treatment cycles 1, 2 and 3 respectively using 30% reduction of headache days compared with baseline as responder rate [12]. OnabotulinumtoxinA was also significantly more effective in reducing a number of secondary efficacy variables including disability. The Headache Impact Test (HIT)-6 score was reduced by 4.8 by onabotulinumtoxinA compared with 2.4 by placebo (*P* < 0.001). Post-hoc analyses demonstrated that headache intensity was reduced in non-responders (patients with less than 50% reduction in headache frequency) [13] and that quality of life was still improved after 1 year of treatment [14]. OnabotulinumtoxinA was well tolerated, the most common adverse events compared with placebo were neck pain (6.7% vs. 2.2%), muscular weakness (5.5% vs. 0.3%), eyelid ptosis (3.3% vs. 0.3%) and injection-site pain (3.2% vs. 2.0%). Discontinuation rates due to adverse events were low (3.8% vs. 1.2%). A secondary analysis of the PREEMPT study investigating the subgroup of patients (1005 out of 1384) who received all 5 treatment cycles demonstrated continued efficacy and tolerability of onabotulinumtoxinA [15].

A subgroup analysis of 904 patients with medication overuse from the PREEMPT studies found similar effect in patients with and without medication overuse [16]. When evaluating these data it should be noted that they were obtained from post hoc analysis [17]. The same was found in a UK study comparing efficacy of onabotulinumtoxinA administered according to PREEMPT in 219 patients with and 215 patients without medication overuse [18].

The good safety and tolerability profile of onabotulinumtoxinA was confirmed in a pooled analysis [19] of two phase 2 studies in chronic daily headache [20, 21] and the two PREEMPT studies [4, 5]. Furthermore, a sub-analysis of 513 subjects receiving all 5 treatment cycles in the PREEMPT studies found that adverse events decreased over time [19].

We identified a number of prospective open-label studies evaluating efficacy and safety of onabotulinumtoxinA [22–39]. In general these studies were long-term studies treating CM patients with and without medication overuse according to the PREEMPT protocol. Several of the studies are limited by some methodological flaws [40]. These studies consistently reported positive results regarding both efficacy and tolerability in line with the PREEMPT studies. A UK study including 254 patients reported responder rates of 32% and 47% when response was defined as ≥50% and ≥ 30% reduction in headache days, respectively [30]. A large multinational study including 1160 patients confirmed the positive safety profile of onabotulinumtoxinA [41]. Longest follow-up reported was in the multinational Chronic migraine OnabotulinuMtoxinA Prolonged Efficacy open Label (COMPEL) study [23], which included 716 patients of whom 373 completed the 2 years follow-up.

A randomized controlled study reported better efficacy of acupuncture than of onabotulinumtoxinA and low-dose valproate [33]. Two studies reported comparable efficacy of onabotulinumtoxinA and topiramate [42, 43] with fewer adverse events from onabotulinumtoxinA [43]. The latter is supported by clinical experience.

Further evidence is under way through the REsource utilisation and Patient-reported OutcomeS (REPOSE) studies [44].

#### Clinical guide

The blinding of the PREEMPT studies has been criticized, because injections in the forehead removing wrinkling could cause un-blinding [6, 7]. In an older study 70% and 60% of patients who knew that they had a 50% chance of receiving placebo, correctly guessed that they were treated with active drug after the first and third treatment cycles respectively [20]. According to GRADE (Table 1) the quality of the PREEMPT studies regarding efficacy is moderate due to risk of un-blinding, while it is high regarding safety because of the very positive safety profile. The high placebo response in the PREEMPT studies [11] and the long-term effect of onabotulinumtoxinA [23] speak against placebo being a major factor.

**Table 1:** GRADE evaluation of placebo-controlled studies evaluating efficacy and tolerability of onabotA

Studies (participants)	Outcome	Comparison	Type	Quality	Effect size	GRADE quality of evidence	Direction	Strength	Comment
Dodick (1384)	Headache days	OnabotA 155 U–195 U versus placebo	R	−1	0	Moderate	For	Strong	Quality points deducted for risk of bias (−1)
Diener (2436)	Adverse events	OnabotA 75 U–260 U versus placebo	R	-1	+ 1	High	For	Strong	Quality points deducted for risk of bias (−1). Effect size point added for small frequency of AE

The Dodick paper [11] was a pooled analysis of the Aurora [4] and 2010 Diener [5] studies. The 2014 Diener paper [19] was a pooled analysis of two phase 2 studies in chronic daily headache [20, 21] and the two PREEMPT studies [4, 5]. Type: *R* randomized controlled trial

Prospective open-label observational studies have serious limitations regarding evaluation of efficacy including lack of control group, risk of publication bias and a potentially high placebo-response. However, the results from the studies are quite consistently positive and support the findings from the placebo-controlled PREEMPT studies. In addition they provide important information on efficacy and tolerability of long-term treatment with onabotulinumtoxinA.

Taking the collected evidence from the PREEMPT studies, the observational studies and clinical experience into consideration, the final quality of evidence is considered to be high regarding both efficacy and safety of onabotulinumtoxinA. The effect size in average is considered modest compared with placebo. However, average group differences in efficacy cannot be considered in isolation as this may obscure meaningful individual patient differences. Direction of recommendation for use of onabotulinumtoxinA is for and strength of recommendation is strong. Quality of evidence for comparison of onabotulinumtoxinA with other prophylactic treatments is too low to make final conclusions, but it is possible that the effect size is comparable to that of topiramate but with a better tolerability profile.

#### Final recommendation

OnabotulinumtoxinA is recommended for treatment of patients with CM and considered an effective and well-tolerated treatment. Quality of evidence: high. Strength of the recommendation: strong.

### Clinical question 2: For patients with CM when should onabotulinumtoxinA be offered?

#### Search strategy and results

Among the retrieved studies, we searched for reports evaluating how onabotulinumtoxinA candidates should be selected. We identified the following relevant questions:

*How many prophylactics should have failed before onabotulinumtoxinA is administered?* NICE performed a subgroup analysis of efficacy related to previous treatment with prophylactic medications. They found that onabotulinumtoxinA was equally effective in patients who had previously received one, two or three or more preventive treatments [6]. Based on cost-effectiveness in the UK, NICE recommends that patients should have failed three or more other migraine prophylactics before onabotulinumtoxinA is administered [6].

*Is it possible to predict who will be good responders?* It has been reported that short disease duration [28, 32] and high serum levels of calcitonin gene-related peptide (CGRP) [27] are predictors of good outcome to onabotulinumtoxinA.

#### Clinical guide

Taking costs into consideration, failure of at least three migraine prophylactics before onabotulinumtoxinA is administered is recommended by NICE. However, this may not apply to all countries, and some experts recommend initiation of onabotulinumtoxinA after failure of at least two to three prophylactics, because topiramate is the only other drug with proved efficacy in CM and because of the better tolerability profile of onabotulinumtoxinA. We recommend that patients should have failed (lack of effect or intolerable side-effects) two to three or more other migraine prophylactics before onabotulinumtoxinA is administered. However, in some patients this is not possible due to multiple comorbid disorders, e.g., cardiac disorder, overweight or depression. Some data indicate that patients who are treated earlier have a better response. However, early stage CM may be more likely to undergo spontaneous fluctuations and/or to improve spontaneously than later stage CM. Yet, there are not enough data to select CM patients to be or not to be treated with onabotulinumtoxinA based on clinical or laboratory characteristics.

#### Final recommendation

It is recommended that patients should have failed at least two to three other migraine prophylactics unless contraindicated by comorbid disorders before onabotulinumtoxinA is administered. This recommendation is based on expert opinion.

### Question 3: For patients with CM plus medication-overuse should withdrawal be done before treatment with onabotulinumtoxinA is initiated?

#### Search strategy and results

Among the retrieved studies we searched for reports comparing treatment with onabotulinumtoxinA initiated before as compared with after withdrawal of the overused medication in patients with CM plus medication overuse. We found no such studies.

#### Clinical guide

There is general consensus that CM patients with medication overuse should be withdrawn from the overused medication (detoxified) [6, 17] but not how this should be done. Evidence indicate that detoxification both with onabotulinumtoxinA [16, 18], detoxification with or without oral prophylactics from the start [45], and detoxification with delayed prophylactic treatment after 2 months [46] are all effective. Because of differences in, e.g., study design and patients characteristics, it is not possible to compare efficacy among these studies. Considering that not all patients need prophylactics after detoxification [47], it is recommended to detoxify first with later initiation of onabotulinumtoxinA when possible. This is in accordance with recommendations from (NICE) [6]. If this is not possible, onabotulinumtoxinA can be initiated from the start or even before withdrawal of the overused medication.

#### Final recommendation

In patients with CM and medication overuse, it is preferable to detoxify first with later initiation of onabotulinumtoxinA. If this is not feasible, onabotulinumtoxinA can be initiated from the start or even before withdrawal of the overused medication. This recommendation is based on expert opinion.

### Clinical question 4: How should treatment with onabotulinumtoxinA be administered?

#### Search strategy and results

Among the retrieved studies, we searched for reports evaluating administration of onabotulinumtoxinA.

The PREEMPT injection protocol, i.e. injection of 155 U–195 U administered to 31–39 sites every 12-weeks as previously described, set the standard for treatment with onabotulinumtoxinA in CM. It has been argued that an injection paradigm customized to the individual patient would be preferable [48]. However, we found no studies comparing the PREEMPT protocol with an alternative protocol.

Treatment with 155 U and 195 U has not been compared head to head. In an open label prospective study, Negro et al. [35] reported higher effect of 195 U than of 155 U given in an earlier study from the same study group.

#### Clinical guide

The PREEMPT injection protocol should be followed, since it is the only protocol that has proved efficacy of onabotulinumtoxinA. It is possible that 195 U is more effective than 155 U. The higher dose could be considered, if the patient does not respond to 155 U.

#### Final recommendation

OnabotulinumtoxinA should be administered according to the PREEMPT injection protocol. This recommendation is based on evidence from the PREEMPT trials.

### Clinical question 5: When can a de novo patient be considered non-responder to onabotulinumtoxinA?

#### Search strategy and results

Among the retrieved studies, we searched for reports evaluating when patients previously naïve to onabotulinumtoxinA can be considered non-responders.

In the PREEMPT studies responders were defined as patients with at least 50% reduction in headache days per month, while NICE defined responders as patients having at least 30% reduction in headache days per month [6]. Like in other chronic pain conditions [49], most headache experts regard a 30% reduction in headache days for clinically relevant in a complicated disorder as CM [12, 50]. Khalil et al. [30] suggested that responders should be defined as either 50% or more reduction in headache or migraine days or doubling of headache-free days (“crystal clear days”) provided that there was at least 3 headache-free days at baseline. Other experts consider a < 30% reduction in headache days as response, if it is accompanied by improvement of other variables such as reduction in headache intensity or improvement of quality of life [50].

A pooled sub-analysis of the PREEMPT studies reported that among onabotulinumtoxinA treated patients 49.3, 11.3 and 10.3% of the patients responded for the first time (50% responders) during treatment cycles 1, 2 and 3 respectively [12]. These data were not tested against placebo. The authors suggested that patients should be offered 2–3 treatment cycles before being categorised as non-responders. Prospective open-label studies reported increased efficacy over 5–9 treatment cycles [35, 38]. NICE recommends that treatment should be stopped if the patient does not respond with at least 30% reduction in headache days per month after two treatment cycles [6].

#### Clinical guide

Response to onabotulinumtoxinA should be continuously monitored by headache calendars. The definition of responders to onabotulinumtoxinA is important for daily clinical practice, but there are currently no convincing data supporting one definition over another. We recommend the use of the definition most easy to use, i.e. that patients should be defined as non-responders, if they have less than 30% reduction in headache days during the first month after treatment with onabotulinumtoxinA compared with the month before first treatment. However, other factors such as headache intensity, disability and patient preferences should also be considered. Treatment should be stopped if the patient does not respond to onabotulinumtoxinA during the first 2–3 treatment cycles (negative stopping rule).

#### Final recommendation

It is recommended that patients should be defined as non-responders, if they have less than 30% reduction in headache days per month during treatment with onabotulinumtoxinA. However, other factors such as headache intensity, disability and patient preferences should also be considered. Treatment should be stopped, if the patient does not respond to the first 2–3 treatment cycles. This recommendation is based on expert opinion.

### Clinical question 6: How should responders to onabotulinumtoxinA be managed over time?

#### Search strategy and results

Among the retrieved studies, we searched for reports investigating when treatment with onabotulinumtoxinA should be stopped once started and how the patients should be managed over time. We found no such studies.

#### Clinical guide

Response to onabotulinumtoxinA should be continuously monitored by headache calendars, where patients record headache days and intake of acute headache medications. Available evidence indicate that electronic calendars, accessible via mobile technologies, may ensure better data recording [45].

There is no generally accepted agreement on from which time point’s response to onabotulinumtoxinA should be evaluated during continued treatment. We suggest that the 4 weeks before and 4 weeks after each treatment cycle should be compared.

NICE recommends that treatment with onabotulinumtoxinA is stopped if the patient has reverted to episodic migraine for three consecutive months [6] (positive stopping rule). This may be impractical in many clinics, because it can be impossible to offer the patient a fast appointment if migraine reverts to CM due to lack of resources. The delay may result in worsening of headache and decreased quality of life [50]. The natural fluctuations occurring over time in CM [51] should also be taken into account. It has been proposed that onabotulinumtoxinA is stopped only in patients with a reduction to less than 10 headache days per month for 3 months given that those with a higher frequency carry a higher risk of relapse to CM [7]. Others have recommended that only in those subjects who are stable responders to onabotulinumtoxinA for at least 1 year, the extension of the inter-injection interval may be a responsible strategy to verify whether the improvement is a long-lasting remission of the disease or attenuation of symptoms due to onabotulinumtoxinA treatment [40].

We recommend that patients are re-evaluated 4–5 months after stopping onabotulinumtoxinA to make sure that the patient has not returned to CM.

#### Final recommendations

It is recommended to evaluate the response to continued treatment with onabotulinumtoxinA on the basis of headache calendars by comparing the 4 weeks before and 4 weeks after each treatment cycle. We recommend to stop treatment in patients with a reduction to less than 10 headache days per month for 3 months. However, other factors such as headache intensity, disability and patient preferences should also be considered. Patients should be re-evaluated 4–5 months after stopping onabotulinumtoxinA to make sure that the patient has not returned to CM. These recommendations are based on expert opinion.

## Discussion

OnabotulinumtoxinA is recommended as an effective and well-tolerated treatment of CM. Patients should preferably have tried 2–3 other migraine prophylactics before start of onabotulinumtoxinA. However, many CM patients bear a considerable load of co-morbidities [52]. This can make it challenging to choose oral prophylactics, since many of these are contraindicated in the presence of, e.g., cardiovascular disease, depression or obesity. Treatment with oral prophylactics is often further complicated by poor tolerability, resulting in low persistence to oral prophylactic treatment in migraineurs [53]. If the patient has some, but insufficient, effect of oral prophylactics, these can go hand in hand with onabotulinumtoxinA if needed.

Management of concomitant medication overuse is controversial and differs considerably among headache centres and countries. At present we have no robust data that favours one approach over another. We recommend that patients with medication overuse should be withdrawn from the overused medication before initiation of onabotulinumtoxinA if feasible, if not onabotulinumtoxinA can be initiated from the start or before withdrawal.

Before the PREEMPT studies, onabotulinumtoxinA was administered according to highly variable protocols in the published reports, e.g. with different doses, number of injection sites and intervals between treatment cycles, resulting in inconsistent results. We recommend that onabotulinumtoxinA is administered according to the PREEMPT injection protocol, i.e. injecting 155 U–195 U to 31–39 sites every 12-weeks, because this is the only treatment paradigm that has scientifically proved to be effective. It is possible that 195 U is more effective than 155 U. The higher dose could be considered, if the patient does not respond to 155 U.

The success of treatment depends not only on how onabotulinumtoxinA is administered but also on how the patient is treated in other aspects. Patients should be educated about their condition and how onabotulinumtoxinA is administered. Patients should be given realistic expectations, i.e. they should be told that the treatment may be able to reduce frequency and intensity of their migraine, but that it does not cure migraine. They should be told that effect usually is seen within 3–7 days, that the effect may wear off before the next treatment cycle, and that response should be evaluated by continued use of headache calendars.

Evaluation of response to migraine treatment is complex, because it involves not only frequency but also severity of headaches, tolerability of the treatment, disability and patient preferences. All of these factors should be taken into consideration when evaluating the response to onabotulinumtoxinA. However, there are no robust data showing which of these variables that best quantifies response to onabotulinumtoxinA. Furthermore, it is impractical for the patients to have to record many variables over a prolonged period of time. We therefore recommend that the simplest measure, headache days, is used as a minimum to monitor response. Other measures such as HIT-6 can be added. We recommend that patients are defined as non-responders, if they have less than 30% reduction in headache days per month during treatment with onabotulinumtoxinA, and that treatment should be stopped, if the patient does not respond to the first 2–3 treatment cycles.

For initial responders to onabotulinumtoxinA, continued evaluation of response is complicated by the fact that the effect of treatment wears off after some time, typically 2–3 months, so which time points should the evaluation be based on? To complicate it more, most responders will not fulfil the criteria for CM when successfully treated, because they will not have had CM for more than 3 months when they show up for the next treatment cycle. So when should treatment be stopped? There are no robust data to guide us on these questions. We recommend that response to continued treatment with onabotulinumtoxinA is evaluated by comparing the 4 weeks before and 4 weeks after each treatment cycle. It is recommended that treatment is stopped in patients with a reduction to less than 10 headache days per month for 3 months and that patients are re-evaluated 4–5 months after stopping onabotulinumtoxinA to make sure that the patient has not returned to CM.

Cost-effectiveness of treatment with onabotulinumtoxinA is an important issue both for the individual and for society. NICE have calculated that treatment with onabotulinumtoxinA is cost-effective, if certain inclusion criteria and stopping rules are adhered to [6]. Likewise, reports from Italy [54], the US [55] and UK [56, 57] claim onabotulinumtoxinA to be cost effective. However, due to the highly variable health care systems in the European countries, it is not possible for us to give general evaluations on the cost-effectiveness of onabotulinumtoxinA.

## Conclusions and recommendations for future research

The experts in the present panel are confident that onabotulinumtoxinA has an important role in the management of CM provided that the recommendations in this guideline are followed. However, only the first of the clinical questions in this guideline, regarding efficacy and tolerability of onabotulinumtoxinA, could be answered on the basis of scientific evidence.

There is a need for studies investigating the role of onabotulinumtoxinA in relation to other prophylactics, including the future calcitonin-gene-related-peptide receptor antagonists, and in relation to withdrawal of medication overuse. Furthermore there are important issues to be better analysed in relation to management with onabotulinumtoxinA over time, including optimal definition of de novo non-responders to onabotulinumtoxinA and even more challenging to decide when and how onabotulinumtoxinA should be considered to be tapered off in the long term management of initial responders.

Future research regarding the treatment of CM with onabotulinumtoxinA may further improve the management of this highly disabling disorder.

## Abbreviations

CM: Chronic migraine
GRADE: Grading of Recommendations, Assessment, Development and Evaluation
NICE: National Institute for Health and Care Excellence
PICO: Patients; Intervention; Comparison and Outcome
PREEMPT: Phase 3 Research Evaluating Migraine Prophylaxis Therapy
